# Community-acquired Methicillin-resistant Staphylococcus aureus among Military Recruits

**DOI:** 10.3201/eid1005.030604

**Published:** 2004-05

**Authors:** Craig E. Zinderman, Byron Conner, Mark A. Malakooti, James E. LaMar, Adam Armstrong, Bruce K. Bohnker

**Affiliations:** * Navy Environmental and Preventive Medicine Unit–Two, Norfolk, Virginia, USA; † Navy Environmental Health Center, Portsmouth, Virginia, USA; ‡ Naval Medical Center, Portsmouth, Virginia, USA

**Keywords:** Military medicine, antimicrobial drug resistance, bacterial drug resistance, methicillin resistance, infectious skin diseases, community acquired infections, soft tissue infections, cellulitis, communicable disease

## Abstract

We report an outbreak of 235 community-acquired methicillin-resistant Staphylococcus aureus (MRSA) infections among military recruits. In this unique environment, the close contact between recruits and the physical demands of training may have contributed to the spread of MRSA. Control measures included improved hygiene and aggressive clinical treatment.

Methicillin-resistant *Staphylococcus aureus* (MRSA) was first recognized in the 1960s and has since become a well-known cause of nosocomial infections (*1*). Recently, MRSA has been reported with increasing frequency outside healthcare settings (*2*–*5*). Community-acquired outbreaks have been reported in a variety of populations, including prison inmates (*3*,*4*), players of contact sports (*6*,*7*), children in daycare (*8*), and crewmembers of a naval ship (*9*). These groups do not possess the risk factors traditionally associated with MRSA infection, namely, recent hospitalization, dialysis, residence in a long-term care facility, or intravenous drug use (*1*,*2*). We report an outbreak of community-acquired MRSA infections among recruits at a large military training facility in the southeastern United States.

## The Study

**Figure 1:**
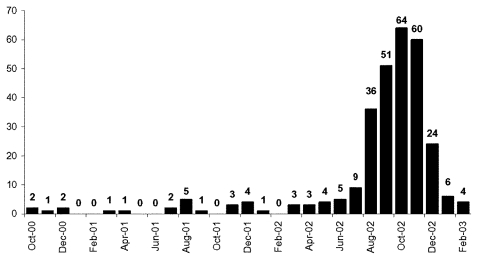
Methicilin-resistant *Staphylococcus aureus* cases in recruits.

**Figure 2:**
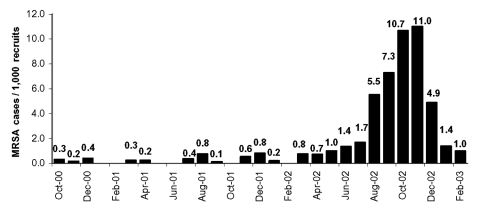
Methicilin-resistant *Staphylococcus aureus* (MRSA) incidence.

The training facility where the outbreak occurred had a recruit population that fluctuated from 3,500 to 7,000. A case-patient was defined as a recruit with a clinically recognized skin or soft-tissue infection and a positive MRSA culture from the site of infection. Laboratory records showed that from October 2000 to July 2002, 47 culture-confirmed MRSA infections occurred among recruits (Figure 1). During this period, the monthly incidence of MRSA did not exceed two cases per 1,000 recruits (Figure 2). However, from August to December 2002, 235 MRSA cases occurred. During the outbreak period, the monthly incidence rates ranged from 4.9 to 11 cases per 1,000 recruits.

Of the case-patients, 209 (89%) were men. This percentage paralleled the overall male recruit population in 2002 (88% male). Although information on the specific age of infected recruits was not available, all recruits at this facility were 17–25 years of age. Most infections occurred on an extremity (73.7%), most commonly the lower leg (16.0%) and the knee (13.9%) (Table).

**Table:** Anatomic site of MRSA infection^a^

Site^b^	No. patients (%)

Lower extremity	86 (44.3)
Thigh/hip	15 (7.7)
Knee	27 (13.9)
Leg	31 (16.0)
Ankle	2 (1.0)
Foot	11 (5.7)
Upper extremity	57 (29.4)
Axilla	8 (4.1)
Arm	20 (10.3)
Elbow	13 (6.7)
Forearm	7 (3.6)
Hand	9 (4.6)
Head	4 (2.1)
Face	4 (2.1)
Neck	3 (1.5)
Torso	3 (1.5)
Back	9 (4.6)
Buttocks	12 (6.2)
Inguinal	1 (0.5)
Genital	4 (2.1)
Urine	1 (0.5)
Sputum	1 (0.5)
Tissue, unspecified	9 (4.6)


^a^MRSA, methicillin-resistant *Staphylococcus aureus*. ^b^Site unknown for 41 patients.

**Figure 3:**
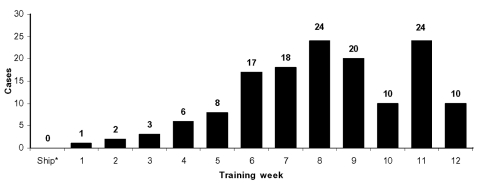
Methicilin-resistant *Staphylococcus aureus* cases by week of training. *Recruits arrive at the facility during ship week and undergo medical and administrative in-processing.

To investigate what aspects of training might be associated with transmission, cases were sorted by week of training when illness was diagnosed (Figure 3). Data on training week was available for 143 (61%) of the outbreak patients. The rise in cases during weeks 1–5 suggests that transmission increased with time in training. Of the cases, 86% occurred during weeks 6 to 12 but did not seem to be associated with any single event. Moderate increases occurred during weeks 6 and 7 (rifle range training) and week 11, which included the “crucible,” a 54-hour strenuous field exercise and final test before graduation. These weeks include important milestones for recruits, and some may have delayed seeking medical care until after completing these steps.

Medical records from 20 patients were randomly selected and reviewed during the investigation. These patients included 18 men and 2 women, 17–24 years of age. The diagnoses included abscesses (15 patients), cellulitis (2 patients), and folliculitis (3 patients). The antimicrobial agents most commonly prescribed for initial treatment were dicloxacillin (*6*), levofloxacin (*5*), and ciprofloxacin (*4*). No patients had a history of hospitalization within the previous year, although one patient had been treated with levofloxacin for pneumonia 2 weeks before.

Nasal screening was conducted to identify carriers and determine the colonization rate among staff members permanently assigned to the training facility. Anterior nasal swabs were obtained from 874 workers who had direct contact with recruits, including medical, dental, and laboratory personnel, drill instructors, barbers, and other ancillary staff. Of these, 24 (2.7%) were colonized with MRSA.

Through interviews with healthcare providers, laboratory personnel, and recruits, investigators found that most patients did not display established risk factors for MRSA (history of chronic medical conditions, hospitalization or surgery within the previous year, history of drug use, or recent use of an antimicrobial agent). Also, the MRSA isolates were sensitive to many commonly used outpatient antimicrobial agents, including trimethoprim/sulfamethoxazole and clindamycin.

No recent lapses in recruit hygiene training or practices had occurred. Recruits were afforded daily time for showering, cleaning, and personal hygiene. However, this time was limited, perhaps leading to deficient hygiene practices among some recruits (i.e., inadequate showering, infrequent handwashing, sharing towels and other personal items).

In November 2002, facility personnel implemented an array of control measures with an emphasis on improving hygiene and treatment regimens. Based on existing recommendations for preventing MRSA transmission in healthcare settings (*10*), antibacterial soaps and hand sanitizers were placed at all recruit sinks, and investigators recommended that hand washing be conducted as frequently as possible. All recruits were issued personal bottles of antibacterial hand sanitizer for use when soap and water were not readily available. Daily showers of adequate duration were enforced, and sharing personal items such as towels and razors was prohibited.

In addition, local healthcare providers were alerted to the presence of MRSA among recruits. Culturing of lesions was encouraged. Patients were treated with the following regimen aimed at eliminating both MRSA infection and nasal carriage: oral rifampin and minocycline for 10 to 14 days, nasal mupirocin twice daily for 10 days, and Hibiclens washes. (Trimethoprim/sulfamethoxazole could be substituted for minocycline.) Finally, preventive medicine staff conducted biweekly surveillance for MRSA cases by using laboratory records.

The outbreak ended in December 2002, shortly after interventions were implemented. The actual number of cases as well as the incidence (cases per 1,000 recruits) declined by more than half in December 2002 and decreased further in January and February 2003 (Figure 1, Figure 2).

## Conclusions

This large outbreak demonstrates the threat of MRSA in a close-contact environment such as recruit training. Our findings are consistent with community-acquired, rather than nosocomial, MRSA infection (*1*–*3*,*6*). MRSA is spread by direct contact, most often through the hands of an infected or nasally colonized person (*3*). Several recent community-acquired MRSA outbreaks have involved comparable close-contact environments (*3*,*8*,*9*). Spread of community-acquired MRSA has also been associated with prolonged physical contact between sports participants (*6*,*7*). Activities such as hand-to-hand combat training, life-saving, and team skill-building exercises involve similar physical contact between recruits.

The physical nature of recruit training is another factor that may have contributed to this outbreak. Recruits often have minor cuts or abrasions that increase the risk of developing skin infections. Such injuries would be expected during physically demanding activities such as running, hiking, and negotiating obstacle courses. Indeed, most MRSA infections occurred on exposed surfaces such as arms, legs, and knees.

The growth and transmission of methicillin-sensitive *S. aureus* (MSSA), and accordingly MRSA, are increased in humid environments (*11*). The number of cases increased during warmer months, a time when recruits have more exposed skin surfaces. This may increase their risk for superficial wounds as well as contact with other recruit’s skin surfaces. Furthermore, some recruits reported that their infections started as insect bites, also a seasonal problem.

The outbreak was unlikely to have originated from a single source. Cases occurred throughout the facility and were not localized to recruits who had contact with a particular instructor or other staff member. Further, the percentage of staff members at the facility who were found to be carriers was small and consistent with the 2%–3% MRSA carriage rates found in recent studies (*9*,*12*,*13*). Although how much contact these carriers routinely had with recruits is unclear, this small number of patients was not likely a major factor in spreading MRSA across so many different groups of recruits. In fact, the growing prevalence of MRSA in the general population (*1*,*2*,*12*,*14*) is an important concern because recruits may enter the military already colonized.

Maintaining good hygiene and avoiding contact with open skin lesions are the primary means to prevent the spread of MRSA infections (*3*,*7*). Although no recent changes in recruit hygiene had occurred that were directly responsible for the outbreak, the hygiene deficiencies noted in some recruits combined with the increased prevalence of MRSA were important contributing factors. Control measures were instituted to improve hygiene, including frequent hand washing and the use of antibacterial hand sanitizers. Similar measures have been implemented to control MRSA outbreaks in comparable settings (*3*,*6*,*7*,*15*).

Before August 2002, healthcare providers did not routinely obtain bacterial identification and sensitivities on skin infections, possibly delaying effective treatment in some cases. Once aware that MRSA was prevalent among recruits, healthcare providers improved treatment by culturing skin lesions whenever possible and prescribing appropriate antimicrobial agents for MRSA infections. Thus, the primary interventions used were recommending improved hygiene practices for recruits and implementing aggressive clinical protocols. These control measures, along with the onset of cooler weather, likely played important roles in ending the outbreak.

This outbreak occurred in a previously healthy military training population and was associated with close contact, limited opportunity for practicing good personal hygiene, warm weather, and physical stress. Reducing MRSA infections was related to implementing interventions to improve personal hygiene, aggressive evaluation and treatment of people with soft tissue injuries and infections, and cooler weather.
